# The role of death receptor 3 in the biological behavior of hepatocellular carcinoma cells

**DOI:** 10.3892/mmr.2014.2858

**Published:** 2014-11-04

**Authors:** YOU CHENG ZHANG, LIU QING GUO, XIAO CHEN, GEN NIAN WANG, RI NI, MAN CAI WANG, FENG XIAN WEI

**Affiliations:** 1 Department of General Surgery, Lanzhou University Second Hospital, Lanzhou, Gansu 730030, P.R. China; 2 Hepato-Biliary-Pancreatic Institute, Lanzhou University Second Hospital, Lanzhou, Gansu 730030, P.R. China; 3 Gansu Provincial-Level Key Laboratory of Digestive System Tumors, Lanzhou, Gansu 730030, P.R. China

**Keywords:** hepatocellular carcinoma, death receptor 3, RNA interference, apoptosis, caspase3, apoptosis antigen-3 ligand

## Abstract

Death receptor 3 (DR3) belongs to the tumor necrosis factor (TNF) receptor superfamily, primarily found in lymphoid tissues. Reports have determined that DR3 may also be distributed in numerous types of tumors. Therefore, it is thought that DR3 may have an important role in the process of tumorigenesis. The aim of the present study was to observe the effect of silencing DR3 expression on hepatocarcinoma cell growth, apoptosis and invasion in order to elucidate the role of DR3 in tumor development. The hepatocarcinoma cell lines (HepG2, Huh7, SMMC7721 and Bel-7402) and normal human liver cells (HL-7702) were transfected with three stealth RNA interference (RNAi) sequences that target the DR3 gene. Reverse transcription quantitative polymerase chain reaction was used to detect the expression levels of DR3 in hepatocarcinoma cell lines and normal liver HL-7702 cells. MTT assay and flow cytometry (FCM) were used to determine the rates of cell proliferation and apoptosis, respectively. Following silencing of the DR3 gene, western blot analysis was used to determine the protein expression of P53, Fas, Caspase8, nuclear factor kappa-light-chain-enhancer of activated B cells (NF-κB) and Caspase3. DR3 messenger RNA (mRNA) expression in hepatocarcinoma cell lines was significantly increased compared with that in the normal liver cell line. Three targeted DR3 gene small interfering RNAs significantly inhibited DR3 gene expression in Bel-7402 cells at the nucleic acid level. AF02670.1_stealth_883 and cocktail demonstrated the most efficient inhibition of DR3 gene expression at 48 and 72 h following transfection, with mRNA inhibition rates of 89.46 and 92.75%, and 90.53 and 94.25% (P<0.01), respectively. Cell viability was significantly reduced by AF02670.1_stealth_883 and RNAi cocktail at 24, 48 and 72 h following transfection. The inhibition rates of cell proliferation were 50.76 and 61.76% (P<0.05) at 72 h following transfection. FCM revealed that AF02670.1_stealth_883 and RNAi cocktail also induced apoptosis in Bel-7402 cells at 72 h following transfection. Reduction of NF-κB and P53 levels was observed (P<0.05) in Bel-7402 cells following DR3 silencing, whereas levels of Fas, Caspase3 and Caspase8 were markedly elevated (P<0.05). DR3 expression levels in hepatocellular carcinoma cells were significantly higher than those in normal cells. DR3 silencing effectively inhibited proliferation and invasion of hepatocellular carcinoma cells *in vitro*. However, silencing of the DR3 gene affect levels of apoptosis antigen-3 ligand in cells, therefore indicating that it may be involved with other pathways that regulate apoptosis in HCCs. In conclusion, the results of the present study indicated that DR3 may be a promising therapeutic target molecule for further study of hepatocellular carcinoma gene therapy.

## Introduction

Hepatocellular carcinoma (HCC) is the sixth most prevalent neoplasm in the world, with the number of reported cases increasing annually. The highest incidence areas are Asia and Africa, of which 80% of cases occur in Asian countries (1). For these countries, HCC malignant tumors are the third most common type of cancer (2). At present, surgical resection, local ablation therapy and liver transplantation are considered fundamental and effective treatments for HCC. However, only ~20% of diagnosed patients benefit from surgical treatment options as the majority of cases HCC are diagnosed in the late stages of tumor progression or the patients have underlying symptoms of cirrhosis; therefore, for numerous patients it is too late for surgery to be effective. In addition, for these cases, radiotherapy and chemotherapy are not curative and can only provide relief of symptoms (3). It has been speculated that HCC occurs due to the synergy of multiple gene abnormalities. Therefore, the discovery of a key regulatory gene for HCC carcinogenesis would provide a new target for HCC therapies.

In 1996, death receptor 3 (DR3), a member of the tumor necrosis factor receptor (TNFR) superfamily, was discovered. DR3 is a type II transmembrane protein containing 417 amino acids and its gene was found to be localized to 1p36.3. DR3, similarly to other TNFR family proteins, has a proteolytic function of an amino acid residue segment on a protein called the death domain in the cytoplasm. The DR3 protein also has a high degree of homology with TNFR-1 and Fas, allowing it to pass on apoptotic signals to downstream pathways, initiating apoptosis (4–6). In addition, DR3 can also activate the expression of nuclear factor kappa-light-chain-enhancer of activated B cells (NF-κB), allowing NF-κB to translocate to the nucleus and activate transcription (7). DR3 was found to be primarily distributed in lymphoid tissues, including the spleen, thymus and peripheral blood lymphocytes (lymphocytes, natural killer cells and macrophages) (8,9). Therefore, previous studies have mainly focused on the involvement of DR3 in immune adjustment, where it was reported to promote the occurrence and development of numerous inflammatory diseases, including inflammatory bowel disease and arthritis (10–14). In addition, DR3 was found to be located in numerous tumor types (15–17). Previously, it was reported that lipeol, at the appropriate dosage, inhibited hepatocarcinoma cell proliferation and apoptosis, as well as reduced the high expression of DR3 (18). Therefore, it has been hypothesized that DR3 may have an important role in the development and progression of HCC. The aim of the present study was to observe the effect of silencing DR3 expression on hepatocarcinoma cell growth, apoptosis and invasion in order to elucidate the role of DR3 in tumor development, and therefore provide a theoretical target for HCC therapies.

## Materials and methods

### Cell culture

The human hepatocarcinoma cell lines Bel-7402, SMMC-7721, Huh7 and HepG2, and normal human liver HL-7702 cells, were purchased from the cell bank of the Chinese Academy of Science (Shanghai, China). The cells were cultured in RPMI-1640 medium (Gibco-BRL, Carlsbad, CA, USA) and DMEM medium (Gibco-BRL) containing 10% fetal bovine serum (FBS; Invitrogen Life Technologies, Carlsbad, CA, USA) in a humidified atmosphere of 5% CO_2_ at 37°C.

### Design and synthesis of Stealth RNA interference (RNAi) small interfering RNA (siRNA) targeting the DR3 gene sequence

Sequences of the three synthesized oligonucleotides were: AF026070.1_stealth_880 sense 5′-UUCUCACUGCUGUCAGGAGGUGCUA-3′ and anti-sense 5′-UAGCACCUCCUGACAGCAGUGAGAA-3′; AF026070.1_stealth_883 sense, 5′-AUCUUCUCACUGCUG UCAGGAGGUG-3′ and anti-sense, 5′-CACCUCCCUGAC AGCAGUGAGAAGAU-3′; AF026070.1_stealth_888 sense, 5′-UGCAGAUCUUCUCAACUGCUGUCAGG-3′ and anti-sense, 5′-CCUGACAGCAGUGAGAAGAUCUGCA-3′. These target sequences were synthesized by Invitrogen Life Technologies and subjected to a Basic Local Alignment Search Tool (www.ncbi.nlm.nih.gov/BLAST.cgi) analysis to ensure that only the DR3 gene was targeted.

### Transfection

To transfect the Stealth RNAi siRNA against DR3 into Bel-7402 and SMMC-7721, Lipofectamine™ RNAiMAX (Invitrogen Life Technologies) was used, and the negative control, the Stealth™ RNAi Negative Control Duplexes (Invitrogen Life Technologies) were used. BLOCK-It™ Alexa Fluor^®^ Red Fluorescent Oligo (Invitrogen Inc, USA) was used to facilitate assessment and optimize the delivery of double-stranded RNA oligonucleotides into Bel-7402 and SMMC-7721 cells, according to the manufacturer’s instructions. Reverse transfection was used to deliver Stealth RNAi siRNA, Red Fluorescent Oligo or negative control duplexes into two cell lines as follows: Lipofectamine™ RNAiMAX complexes were prepared according to the manufacturer’s instructions, cells were seeded at appropriate dilutions and incubated for 24 h to reach 30–50% confluence. The complexes were added to the cells (5×10^5^) and incubated for 48 and 72 h at 37°C in a CO_2_ incubator until ready for gene knockdown assays.

### Nucleic acid assessment

Total RNA was extracted using RNAiso™ Plus (Takara Co., Ltd., Otsu, Japan), following transfection. First strand complementary DNA (cDNA) synthesis and amplification were performed using a two-step reverse transcription quantitative polymerase chain reaction (RT-qPCR) kit (Takara Co., Ltd.) with the Rotor-Gene 6,000 (Qiagen, Hilden, Germany). The reverse transcription reaction was performed at 37°C for 15 min followed by 85°C for 5 min in order to inactivate reverse transcriptase. The reaction volume was 25 μl, with 12.5 μl SYBR^®^ Premix Ex Taq™ II (2X) (Takara Co., Ltd.).

The sequences of primers were: DR3 sense, 5′-GTGTGTCCCCAAGACACCTT-3′ and anti-sense, 5′-GTCTAGGCATGGTTGGCAGT-3′ (GenBank accession no. AF026070.1); Casepase3 sense, 5′-GGTTCATCC AGTCGCTTTGT-3′ and anti-sense, 5′-CGGTTAACCCGGGT AAGAAT-3′ (GenBank accession no. NM_004346.3); Caspase8 sense, 5′-CCAAATGCAAACTGGATGATGAC-3′ and anti-sense, 5′-CTCTTGTTGATTTGGGCACAG AC-3′ (GenBank accession no. NM-001228.4); NF-κB sense, 5′-TTGTGGCCGCCTAAGTGGA-3′ and anti-sense, 5′-ACCACCTTGATCTGGGTAGCACATA-3′ (GenBank accession no. AF018253.1); P53 sense, 5′-GGCCCACTTCACCGTACTAA-3′ and anti-sense, 5′-GTGGTTTCAAGGCCAGATGT-3′ (GenBank accession no. NM_000546.4); apoptosis antigen-3 ligand (Apo-3L) sense, 5′-GAGGAATTCTCAGCCACTGC-3′ and anti-sense, 5′-CCCTCAGTGAACCTGGAAGA-3′ (GenBank accession no. NM_003809.2); and β-actin sense, 5′-AGAGATGGCCACGGCTGCTT-3′ and anti-sense, 5′-ATTTGCGGTGGACGATGGAG (GenBank accession no. NM_001101.3) (Takara Co., Ltd.).

### Protein assessment

Cultured cells were lysed in lysis buffer phenylmethylsulfonyl fluoride (Sangon Biotech, Shanghai, China), and a protein standard curve was used to calculate the density of total protein. Proteins were separated using 10% SDS-PAGE (Sangon Biotech) under denaturing conditions and then transferred to nitrocellulose membranes (Applygen Technologies, Inc., Beijing, China). Membranes were incubated with rat anti-DR3 polyclonal antibody (pAb), rabbit anti-TNF-related weak inducer of apoptosis (TWEAK) pAb, rabbit anti-NF-κB pAb, rabbit Caspase3 monoclonal antibody (mAb), rabbit Caspase8 mAb, rat anti-P53 pAb, rabbit anti-Fas pAb or rat anti-β-actin mAb primary antibodies (all 1:1,000; Abcam, Cambridge, MA, USA), followed by incubation with anti-mouse secondary antibody conjugated to horseradish peroxidase (1:5,000; Amersham Biosciences, Chalfont St. Giles, UK). Immunoreactive proteins were visualized using Poncuar S staining solution (Sangon Biotech), developing liquid and fixing solution (Beyotime Institute of Biotechnology, Shanghai, China), Super ECL Plus (Applygen Technologies Inc. Beijing, China) and a Bio-Rad gel imaging system (Bio-Rad, Hercules, CA, USA).

### Cell proliferation assessment

Following reverse transfection of Bel-7402 cells with Stealth RNAi siRNA targeting the DR3 gene or negative control duplexes in 96-well plates, MTT (Sigma-Aldrich, St. Louis, MO, USA) was added at 24, 48 and 72 h, in order to determine the rates of cell proliferation. The optical density was measured using a UR-4100 plate reader (Fisher Thermo Scientific, Waltham, MA, USA).

### Assessment of apoptosis

To determine the occurrence of apoptosis within 72 h of transfection, cells were harvested using trypsinization (Sigma-Aldrich) and rinsed twice with phosphate-buffered saline (Sangon Biotech). Cells were then centrifuged (4°C, 1,000 ×g) for 10 min. Cells were resuspended in 200 μl binding buffer (Sangon Biotech) and treated with 10 μl Annexin V-fluorescein isothiocyanate (FITC) and 5 μl propidium iodide (PI; both Sigma-Aldrich) for 15 min at room temperature. The rate of apoptosis was then determined using flow cytometry (Epics-XL; Beckman-Coulter, Shanghai, China).

### Invasion assessment

Invasion assessments were performed using a 24-well Transwell chamber (Corning Inc., Corning, NY, USA). Each Transwell chamber was coated with 200 μl Matrigel^®^ (Corning, Inc.), which was pre-diluted into 100 μg/ml with 0.1% FBS). A total of 5×10^5^/ml cells were seeded into the pre-coated wells. The lower parts of the chambers were filled with 200 μl RPMI 1640 medium containing 10% FBS. Following incubation for 24 h, the cells on the upper surface were gently removed using a cotton swab, and the filters were fixed with 95% ethanol for 30 min and stained with 0.1% hexamethylpararosaniline (both Sangon Biotech) for 15 min. The number of cells on the lower surface of the membranes was quantified using a microscope (LX71; Olympus Corp., Tokyo, Japan).

### Statistical analysis

Statistical analysis was performed using the SPSS 16.0 statistical software package (IBM, Armonk, NY, USA). P<0.05 was considered to indicate a statistically significant difference between values.

## Results

### Content, purity and integrity of total RNA in different cell lines

**Figure 1:**
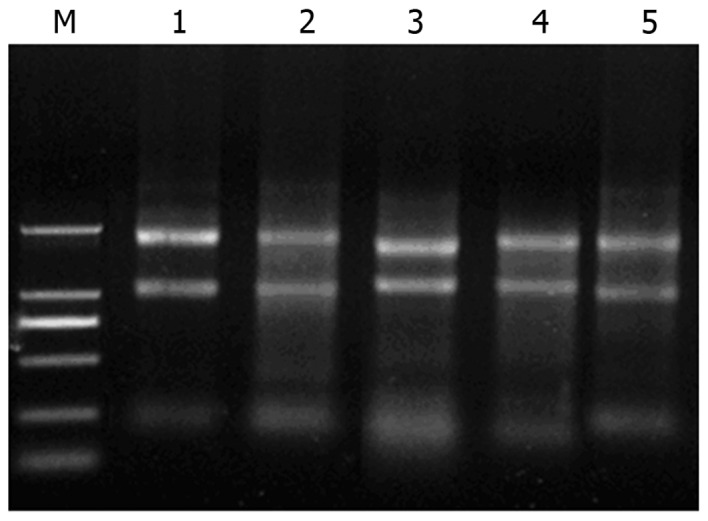
SDS-PAGE for total RNA analysis. Lanes from left to right: M, DL2000 Marker; 1, Huh7; 2, SMMC7721; 3, HepG2; 4, Bel-7402; and 5, HL-7702 cells. In each electrophoresis lane, three bands are observed, with sedimentation coefficients of ribosomal RNA of s=28, 18 and 5, demostrating the high integrity of total RNA.

An ultraviolet spectrophotometer was used to detect the total RNA at the optical density (OD)260/OD280 (R) from the cell lines Huh7, SMMC7721, HepG2, Bel-7402 and HL-7702. The R-values were 1.85, 1.86, 1.95, 1.91, and 2.01, reflecting high levels of purity. The integrity of total RNA is shown in Fig. 1.

### The expression of DR3 messenger RNA (mRNA) in different cell lines

**Figure 2:**
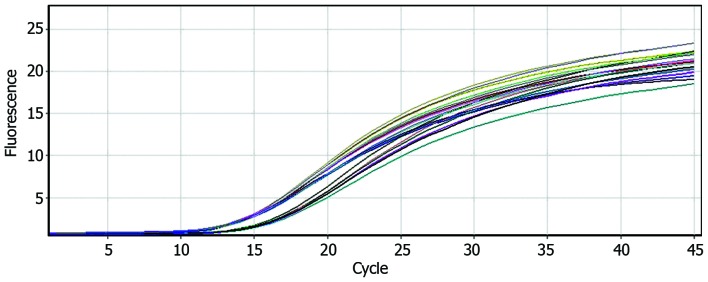
Amplification curve of cDNA from HepG2, Huh7, SMMC7721, Bel-7402 and HL-7702 cells (23 copies demonstrating reliability).

**Figure 3:**
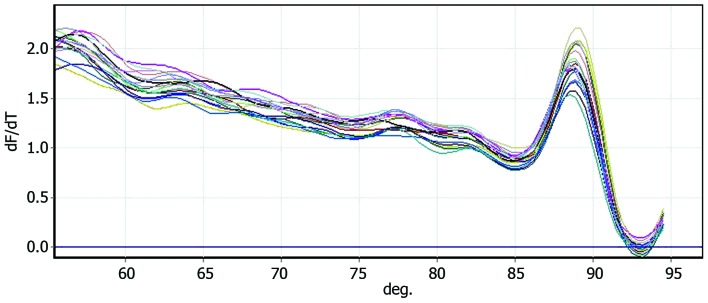
Melting curve of cDNA from HepG2, Huh7, SMMC7721, Bel-7402 and HL-7702 cells (23 copies demonstrating reliability).

Following the extraction of total RNA from each cell line, they were reverse-transcribed into cDNA. To ensure the reliability and accuracy of the results, the target gene-specific primers were amplified at the same time as β-actin (Figs. 2 and 3). The ΔΔCt method was used to calculate the relative expression levels of each DR3 sample (Table I). DR3 expression at the nucleic acid level was detected in multiple strains of hepatocarcinoma cell lines. DR3 expression was significantly higher in hepatocarcinoma cell lines compared to that of normal liver cells. Therefore, it was speculated that DR3 expression may be associated with the occurrence of liver cancer.

### Transfection efficiency and conditions

**Figure 4:**
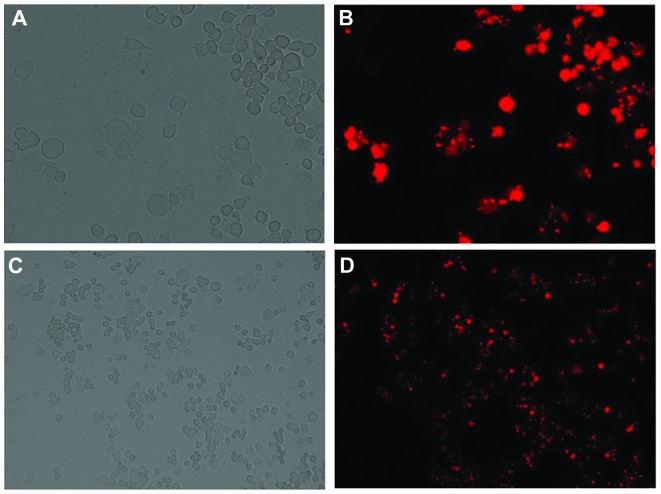
Detection of transfection efficiency following transfection of liver cancer Bel-7402 cells with the Stealth small interfering RNA. 24 h post transfection, the transfection efficiency was >85%, as revealed by (A and C) light and (B and D) fluorescence microscopy. (A and B, magnification, ×400; C and D, magnification, ×100).

Following the determination of transfection efficiency in Bel-7402 cells, it was revealed that when using 30 nM red fluorescent oligo and 5×10^5^/ml Bel-7402 cells, the efficiency reached >85% (Fig. 4). These were therefore the conditions used to perform the subsequent experiments.

### Screening of the most efficient stealth RNAi sequence targeting DR3

**Figure 5:**
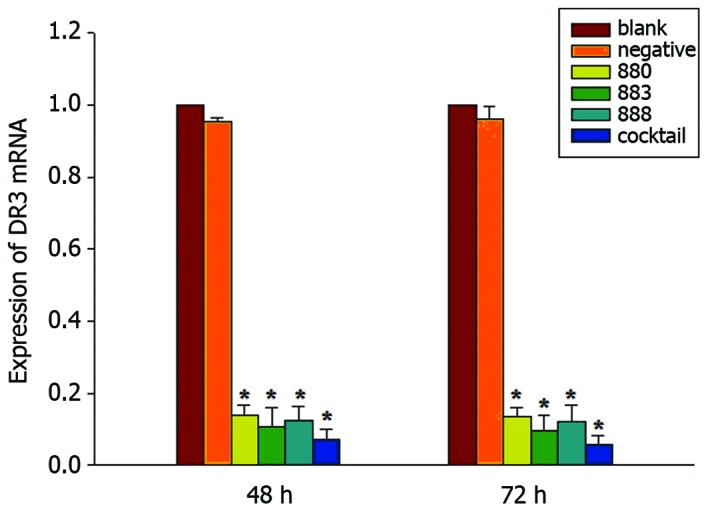
Expression of DR3 messenger RNA following transfection. DR3, death receptor 3; 880, transfected with AF026070.1_stealth_880; 883, transfected with AF026070.1_stealth_883; 888, transfected with AF026070.1_stealth_883; cocktail, transfection with combination of the three small interfering RNAs.

Following transfection of Bel-7402 cells with the stealth siRNA targeting DR3, the RT-qPCR method was used to measure mRNA levels of DR3. A significant decrease in DR3 mRNA levels following DR3 RNAi was observed, while the levels of β-actin remained unchanged. AF02670.1_stealth_883 demonstrated a more potent suppression of DR3 mRNA than AF026070.1_stealth_880 or AF02670.1_stealth_888; however, a cocktail of the three stealth RNAi siRNAs caused the most potent decrease in DR3 mRNA expression. Quantification revealed that AF02670.1_stealth_883 reduced DR3 mRNA by 89.46 and 90.53% of the blank control at 48 and 72 h following transfection, respectively. The cocktail reduced DR3 mRNA expression by 92.75 and 94.25% compared with that of the blank control at 48 and 72 h following transfection, respectively, while the AF026070.1_stealth_880, AF02670.1_stealth_888 and negative control group caused decreases of 86.03, 87.52 and 4.70% at 48h, as well as 86.48, 88.01 and 3.94% at 72 h following transfection, respectively (Fig. 5).

### Cell proliferation assessment

**Figure 6:**
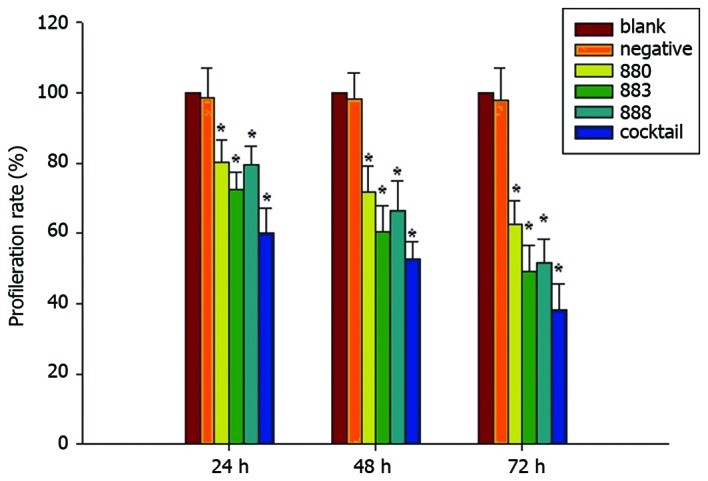
Proliferation rate of Bel-7402 cell line. 880, transfected with AF026070.1_stealth_880; 883, transfected with AF026070.1_stealth_883; 888, transfected with AF026070.1_stealth_883; cocktail, transfection with combination of the three small interfering RNAs.

MTT assays were used to determine the effect of transfection at 24, 48 and 72 h on the proliferation in Bel-7402 cells. Cell viability was reduced significantly following treatment with the individual stealth siRNAs as well as the cocktail against DR3, compared to that of the negative and blank controls (P<0.05) (Fig. 6). The cocktail caused the most potent suppression of proliferation with inhibition rates of 39.86, 47.51 and 61.76% at 24, 48 and 72 h following transfection, with the AF026070.1_stealth_883 siRNA causing the most efficient decrease, with proliferation rates of 27.59, 39.47 and 50.76% of that of the control at 24, 48 and 72 h, respectively (Fig. 6).

### Assessment of apoptosis

**Figure 7:**
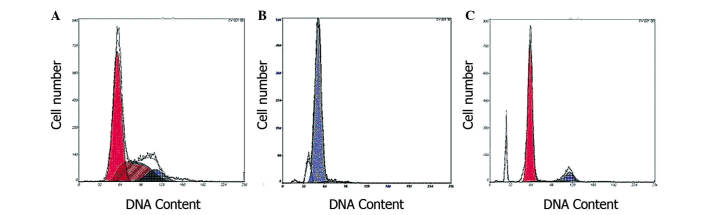
Detection of apoptosis by flow cytometry (48 h). (A) Normal Bel-7402 cell line, %Tot=0.203; (B) AF02670.1_stealth_883 interference, %Tot=8.663; (C) Apoptosis following transduction with cocktail, %Tot=10.425. Tot, total.

**Figure 8:**
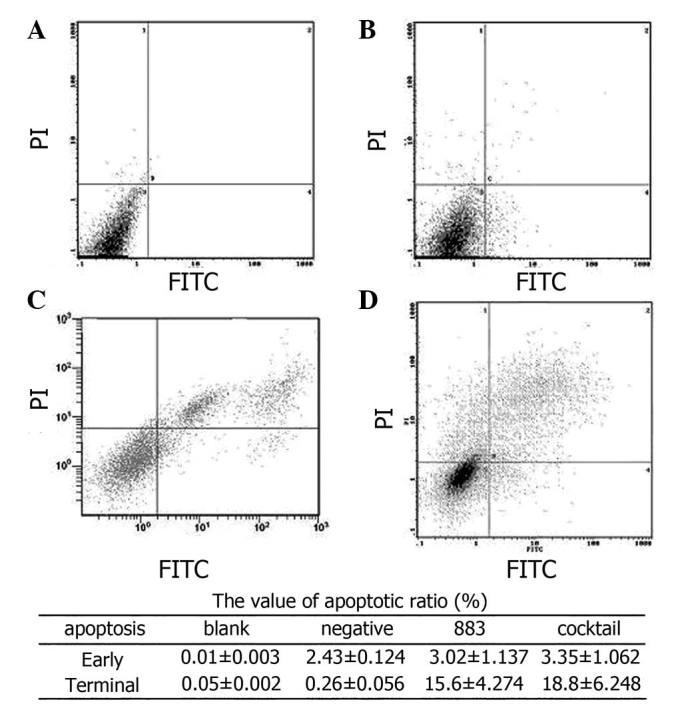
Detection of apoptosis (72 h following transfection) using PI/Annexin V FITC double staining. (A) Liver cancer cell line Bel-7402; (B) negative control; (C) interference of apoptosis by AF026070.1_stealth_883; and (D) interference of apoptosis by cocktail. PI, propidium iodide; FITC, fluorescein isothiocyanate.

To determine the effects of DR3-silencing on apoptosis, a flow cytometric PI single-staining assay was used on Bel-7402 cells following 48 h transfection. PI staining revealed that following AF026070.1_stealth_883 or cocktail treatment, an apoptotic sub-G1 cell population was present, and the apoptotic rates were 8.663 and 10.425% (P<0.05) compared with those of the normal Bel-7402 cells at 0.203% (Fig. 7). In order to further investigate the occurrence of apoptosis, double staining with FITC/PI was used, the results of which demonstrated that following 72 h of transfection with AF026070.1_stealth_883 or cocktail, the proportion of apoptotic cells at the terminal stage was significantly increased compared to those in the negative and blank control groups (Fig. 8).

### Invasion assay

**Figure 9:**
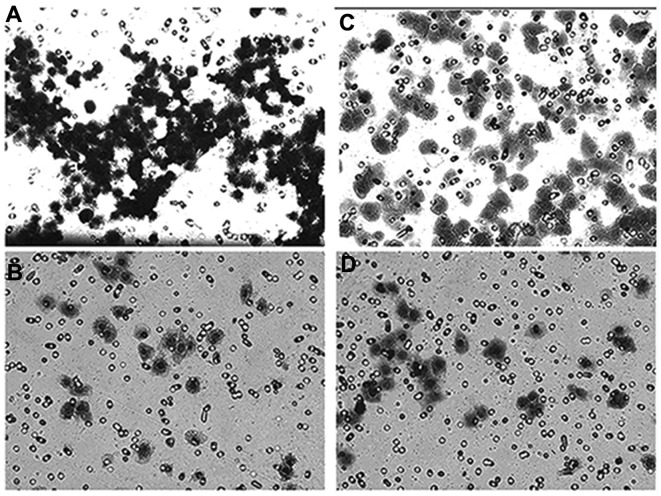
Changes in cell invasive abilities as measured by Transwell assay. (A) Blank; (B) RNA interference with cocktail; (C) negative control; (D) RNA interference with AF026070.1_stealth_883. Magnification, ×100.

The results of the Transwell cell invasion assay are presented in Fig. 9. Following 72 h of transfection by AF026070.1_stealth_883 or cocktail, the invasion ability of Bel-7402 cells was significantly reduced compared with that of the blank and negative controls.

### Protein assessment

Western blot analysis was used to determine the expression levels of several proteins in order to elucidate the mechanisms of DR3 gene silencing-induced apoptosis. Following 72 h of transfection with AF026070.1_stealth_883 or cocktail into Bel-7402 cells, protein levels of P53, Caspase3, Fas, Caspase8, NF-κB, and DR3/TWEAK were detected (Table II). The results revealed significantly reduced expression of P53 and NF-κB (P<0.05), whereas Caspase3, Fas, and Caspase8 were increased in the AF026070.1_stealth_883 and cocktail groups (P<0.05). Levels of DR3/TWEAK showed no significant change.

## Discussion

Due to the rapid progression in the fields of cellular and molecular biology, molecular oncology and other disciplines, the mechanisms of numerous cell signaling pathways and target molecules have been elucidated, therefore allowing for the discovery of targeted oncology drugs such as Sorafenib, which was approved as a first-line drug for the treatment of advanced renal cell carcinoma with dual anti-tumor effects by the US Food and Drug Administration in November 2005. Sorafenib was reported to be able to directly inhibit tumor growth via inhibition of the rapidly accelerated fibrosarcoma/mitogen-activated protein kinase kinase/extracellular signal-regulated kinase signaling pathway. Conversely, Sorafenib also inhibited the activity of several tyrosine kinase receptors associated with angiogenesis, and tumor development, preventing tumor angiogenesis, thereby indirectly inhibiting tumor growth (19–21). Bevacizumab [Avastin, recombinant humanized monoclonal antibody-vascular endothelial growth factor (rhuMAb-VEGF)] was the first approved drug to inhibit tumor revascularization via VEGF. As a synthetic recombinant humanized immunoglobulin G1 monoclonal antibody, bevacizumab can specifically bind to VEGF and form an inhibitory combination of VEGF and endothelial cell surface receptors fms-related tyrosine kinase 1 and kinase insert domain receptor, thereby preventing endothelial cell proliferation and tumor angiogenesis and subsequently inhibiting tumor growth (22–24). Another signal transmitter and activator of the transcription factor 3 [signal transducer and activator of transcription 3, (STAT3)] signaling pathway of tumor necrosis factor (TNF)-related apoptosis-inducing ligand (TRAIL) has also been studied for its involvement in tumor-associated signal transduction pathways (25–27). The results of these studies determined the necessity of understanding the roles of target molecules in cancer cell survival and provide a broad spectrum of molecules and pathways for further study. Although the mechanisms of numerous signaling pathways and their target molecules have been elucidated, resulting in numerous effective therapeutic strategies (28–30), gene therapy for hepatocellular carcinoma has remained ineffective. Therefore, research into finding the key molecules that regulate the growth of hepatocarcinoma cells has become an important task for elucidating the mechanisms of HCC and enhancing the prospects of therapeutic gene therapy.

DR3, a type of death receptor of the TNF superfamily (6), was discovered by the expressed sequence tag database (http://www.ncbi.nlm.nih.gov/nuccore/AF026070.1) from the umbilical vein endothelial cell cDNA library, following screening with TNF in 1996, for having similar applications to a clone of the family members, also known as Apo-3, Trf4/Air2/Mtr4p polyadenylation complex (TRAMP, lymphocyte-associated receptor of death), WSL-1 (28,31–33). DR3 is usually found in organs rich in lymphoid tissue, including the spleen, thymus, small intestine and peripheral blood lymphocytes (6). Apoptosis is the physiological programmed death of cells; a key feature of apoptosis, caspase enzyme cascade reaction, can occur via internal and external triggers, ultimately resulting in the breakdown of DNA, thereby causing karyopyknosis or karyorrhexis (34–37). Apoptosis consists of three main pathways, namely the mitochondrial pathway, the death receptor pathway and the endoplasmic reticulum pathway (38–40). The occurrence of apoptosis via the death receptor pathway occurs when specific cell surface death receptors are triggered by extracellular death signals, which then activate the intracellular mechanism of apoptosis induction. Previous studies on DR3 reported that under pathological conditions, DR3 may cause apoptosis via a downstream death domain (41,42). Studies have demonstrated that the expression of DR3 in the hepatocarcinoma cell lines SMMC-7721 and HepG2 was increased compared to that of normal liver cells; furthermore, the anti-cancer drug lupeol, was shown to have potential in decreasing DR3 expression levels in hepatocellular carcinoma cells (43). In 2006, Gout *et al* (15) reported that DR3 expression in colon cancer tissue was higher than that in adjacent and normal colon tissues and that silencing the gene expression of DR3 reduced colon cancer HT29 cell adhesion and migration capacity, as well as weakened the metastatic potential of HT29 cells (16). In addition, there have been several studies investigating the association between the DR3 and HCC; Jiang *et al* (16) found that DR3 was highly expressed in hepatocarcinoma H3B cells. However, the role of DR3 in the progression of HCC remains to be elucidated; furthermore, it remains to be explained why the high expression of DR3 in tumor cells fails to induce apoptosis.

The aim of the present study was to clarify the role of DR3 in human hepatocarcinoma cells. RNAi siRNAs were used to silence DR3 expression in the hepatocarcinoma cell line Bel-7402. RT-qPCR experiments demonstrated that the three targeted DR3 siRNAs (Stealth siRNA AF026070.1_stealth_880, AF02670.1_stealth_883 and AF02670.1_stealth_888) and cocktail mixtures following transfection for 48 and 72 h effectively inhibited the expression of DR3 mRNA, with a silencing efficiency of >85%; the highest silencing efficiencies, expressed as the inhibitory rate of DR3 mRNA levels, were achieved by AF02670.1_stealth_883 and a cocktail of the three sequences following transfection for 48 and 72 h at 89.46 and 92.75%, and 90.53 and 94.25% (P<0.01), respectively.

MTT assays confirmed that silencing DR3 gene expression significantly inhibited cell proliferation in Bel-7402 cells following transfection with AF026070.1_stealth_880, AF02670.1_stealth_883, AF02670.1_stealth_888 and a cocktail of the three the Stealth™ RNAi siRNAs at 24, 48 and 72 h (P<0.05). The most potent inhibition of cell proliferation was observed with AF02670.1_stealth_883 and cocktail 72 h following transfection, at 50.76 and 61.76% (P<0.05) compared to that of the negative control siRNA, which showed no inhibitory effect on the growth of Bel-7402 cells. Flow cytometry following PI staining and PI/FITC double staining confirmed that DR3-silencing induced apoptosis, and Transwell experiments demonstrated significantly reduced hepatocarcinoma cell invasion. These results indicated that high expression of DR3 in hepatocarcinoma Bel-7402 cells may promote proliferation and inhibit apoptosis.

Western blot analysis revealed that following DR3 silencing, the expression levels of apoptosis-associated proteins, including Fas, Caspase8 and Caspase3, were increased, while the expression of NF-κB was significantly reduced, which was consistent with the MTT results. Protein levels of the mitochondrial transcription factor P53 were also significantly decreased, indicating DR3 may also interact with the mitochondrial pathway to regulate apoptosis. Expression levels of Apo-3L were not significantly altered; therefore, it was hypothesized that Apo-3L does not only bind DR3 in the regulation of tumor cell apoptosis, but acts as a ligand for other regulators of apoptosis in tumor cells that remain to be elucidated.

In conclusion, the results of the present study demonstrated that silencing the expression of DR3 significantly inhibited hepatocarcinoma cell proliferation and invasion, therefore indicating that DR3 promotes proliferation and invasion of HCC, as well as downregulates apoptosis. Overall, the inhibition of DR3 may be a potential target for the treatment of HCC.

Based on the results of the present and previous studies, the next progression of research into DR3-targeted therapies is to construct a targeted small hairpin RNA (shRNA) plasmid vector of the DR3 gene and cultivate stable DR3 silenced hepatocarcinoma cell lines for nude mice experiments *in vitro.* These experiments will aim to compare DR3 −/− and high expressing DR3 liver cancer cell tumorigenicity and metastatic ability. Furthermore, yeast two-hybrid technology, with DR3 as a bait can be used to screen a human liver cDNA library for cDNA that can interact with the protein. Bioinformatics may then be employed to identify positive clones; immunoprecipitation technology may be used to identify the positive clones expressing specific proteins with a specific DNA fragment of combination and precipitation, collected for the purpose of analyzing fragments to identify the DR3-specific binding molecules in liver cancer cells and which initiate the apoptotic program. This may aid in accurately clarifying the molecular biological mechanism of DR3 in the process of development of HCC, and provide theoretical support for molecular targeted therapy of HCC.

## Figures and Tables

**Table I:** Expression of DR3 in different cell lines.

Cell lines	DR3(Ct)	β-actin(Ct)	ΔCt	ΔΔCt	2^−ΔΔCt^
HL-7702	19.5	10.54	8.71	0	1
Hhh7	16.71	10.36	6.35	−2.36	5.134
Bel-7402	16.40	9.74	6.66	−2.05	4.141
SMMC-7721	16.05	8.97	7.08	−1.63	3.095
HepG2	16.03	9.13	6.90	−1.81	3.506

DR3, death receptor 3.

**Table II:** Expression of proteins at 72 h after transfection.

Protein	Blank	Negative	883	Cocktail
DR3	0.472±0.045	0.428±0.037	0.072±0.026	0
Apo-3L	0.884±0.054	0.867±0.062	0.853±0.075	0.850±0.047
NF-κb	0.571±0.051	0.528±0.065	0.293±0.047	0.144±0.038
P53	0.973±0.081	0.892±0.076	0.774±0.068	0.683±0.084
Fas	0.036±0.039	0.041±0.053	0.479±0.062	0.492±0.048
Caspase8	0.029±0.007	0.043±0.012	0.480±0.027	0.503±0.037
Caspase3	0.370±0.035	0.400±0.026	0.538±0.044	0.582±0.030

DR3, death receptor 3; Apo-3L, apoptosis antigen-3 ligand; NF-κb, nuclear factor kappa b. 883, cells in which DR3 was silenced with AF026070.1_stealth_883 RNA; Cocktail, cells in which DR3 was silenced using AF026070.1_stealth_883, −880 and −888 RNA.

## References

[1] Parkin DM, Bray F, Ferlay J, Pisani P (2001). Estimating the world cancer burden: Globocan 2000. Int J Cancer.

[2] Llovet JM, Bruix J (2008). Novel advancements in the management of hepatocellular carcinoma in 2008. J Hepatol.

[3] Bergé M, Bonnin P, Sulpice E (2010). Small interfering RNAs induce target-independent inhibition of tumor growth and vasculature remodeling in a mouse model of hepatocellular carcinoma. Am J Pathol.

[4] Schuster MJ, Wu GY (1997). Gene therapy for hepatocellular carcinoma: progress but many stones yet unturned. Gastroenterology.

[5] Chinnaiyan AM, O’Rourke K, Yu GL (1996). Signal transduction by DR3, a death domain-containing receptor related to TNFR-1 and CD95. Science.

[6] Ashkenazi A, Dixit VM (1998). Death receptor: signaling and modulation. Science.

[7] Croft M (2009). The role of TNF superfamily members in T-cell function and diseases. Nat Rev Immunol.

[8] Kang YJ, Kim WJ, Bae HU (2005). Involvement of TL1A and DR3 in induction of proinflammatory cytokines and matrix metalloproteinase-9 in atherogenesis. Cytokine.

[9] Fang L, Adkins B, Deyev V, Podack ER (2008). Essential role of TNF receptor superfamily 25 (TNFRSF25) in the development of allergic lung inflammation. J Exp Med.

[10] Migone TS, Zhang J, Luo X (2002). TL1A is a TNF-like ligand for DR3 and TR6/DcR3 and functions as a T cell costimulator. Immunity.

[11] Pappu BP, Borodovsky A, Zheng TS (2008). TL1A-DR3 interaction regulates Th17 cell function and Th17-mediated autoimmune disease. J Exp Med.

[12] Meylan F, Davidson TS, Kahle E (2008). The TNF-family receptor DR3 is essential for diverse T cell-mediated inflammatory diseases. Immunity.

[13] Bamias G, Martin C, Marini M (2003). Expression, localization, and functional activity of TL1A, a novel Th1-polarizing cytokine in inflammatory bowel disease. J Immunol.

[14] Warzocha K, Ribeiro P, Charlot C, Renard N, Coiffier B, Salles G (1998). A new death receptor 3 isoform: expression in human lymphoid cell lines and non-Hodgkin’s lymphomas. Biochem Biophys Res Commun.

[15] Gout S, Morin C, Houle F, Huot J (2006). Death receptor-3, a new E-Selectin counter-receptor that confers migration and survival advantages to colon carcinoma cells by triggering p38 and ERK MAPK activation. Cancer Res.

[16] Jiang S, Song MJ, Shin EC, Lee MO, Kim SJ, Park JH (1999). Apoptosis in human hepatocarcinoma cell lines by chemotherapeutic drugs via Fas-dependent and Fas-independent pathways. Hepatology.

[17] Zhang L, Zhang Y, Zhang L, Yang X, Lv Z (2009). Lupeol, a dietary triterpene, inhibited growth and induced apoptosis through down-regulation of DR3 in SMMC7721 cells. Cancer Invest.

[18] Strumberg D (2005). Preclinical and clinical development of the oral multikinase inhibitor sorafenib in cancer treatment. Drugs Today (Barc).

[19] Adnane L, Trail PA, Taylor I, Wilhelm SM (2006). Sorafenib (BAY 43-9006, Nexavar), a dual-action inhibitor that targets RAF/MEK/ERK pathway in tumor cells and tyrosine kinases VEGFR/PDGFR in tumor vasculature. Methods Enzymol.

[20] Rini BI (2006). Sorafenib. Expert Opin Pharmacother.

[21] Willett CG, Boucher Y, di Tomaso E (2004). Direct evidence that the VEGF-specific antibody bevacizumab has antivascular effects in human rectal cancer. Nat Med.

[22] Goodman L (2004). Persistence - luck - Avastin. J Clin Invest.

[23] Bergsland E, Dickler MN (2004). Maximizing the potential of bevacizumab in cancer treatment. Oncologist.

[24] Zhu AX, Blaszkowsky LS, Ryan DP (2006). Phase II study of gemcitabine and oxaliplatin in combination with bevacizumab in patients with advanced hepatocellular carcinoma. J Clin Oncol.

[25] David D, Rajappan L, Balachandran KK (2011). Prognostic significance of STAT3 and phosphorylated STAT3 in human soft tissue tumors-a clinicopathological analysis. J Exp Clin Res.

[26] Roth W, Grund K, Wiestler OD, Schirmacher P (2007). The anti-diabetic drug troglitazone sensitizes colon cancer cells to TRAIL-induced apoptosis by down-regulating FLIP. Verh Dtsch Ges Pathol.

[27] Zhao B, Li L, Cui K, Wang AL (2011). Mechanisms of TRAIL and gemcitabine induction of pancreatic cancer cell apoptosis. Asian Pac J Cancer Prev.

[28] Wilhelm SM, Carter C, Tang L (2004). BAY 43-9006 exhibits broad spectrum oral antitumor activity and targets the RAF/MEK/ERK pathway and receptor tyrosine kinases involved in tumor progression and angiogenesis. Cancer Res.

[29] Herold-Mende C, Steiner HH, Andl T (1999). Expression and functional significance of vascular endothelial growth factor receptors in human tumor cells. Lab Invest.

[30] Kitson J, Raven T, Jiang YP (1996). A death-domain-containing receptor that mediates apoptosis. Nature.

[31] Marsters SA, Sheridan JP, Donahue CJ (1996). Apo-3, a new member of the tumor necrosis factor receptor family, contains a death domain and activates apoptosis and NF-kappa B. Curr Biol.

[32] Bodmer JL, Burns K, Schneider P (1997). TRAMP, a novel apoptosis-mediating receptor with sequence homology to tumor necrosis factor receptor 1 and Fas (Apo-1/CD95). Immunity.

[33] Screaton GR, Xu XN, Olsen AL, Cowper AE, Tan R, McMichael AJ, Bell JI (1997). LARD: A new lymphoid-specific death domain containing receptor regulated by alternative pre-mRNA splicing. Proc Natl Acad Sci USA.

[34] Abou-Alfa GK, Schwartz L, Ricci S (2006). Phase II study of sorafenib in patients with advanced hepatocellular carcinoma. J Clin Oncol.

[35] Butterfield LH (2004). Immunotherapeutic strategies for hepatocellular carcinoma. Gastroenterology.

[36] Butterfield LH (2007). Recent advances in immunotherapy for hepatocellular cancer. Swiss Med Wkly.

[37] Song G, Luo Q, Qin J, Wang L, Shi Y, Sun C (2006). Effects of oxymatrine on proliferation and apoptosis in human hepatoma cells. Colloids Surf B Biointerfaces.

[38] Ashkenazi A, Dixit VM (1998). Death receptors: signaling and modulation. Science.

[39] Hengartner MO (2000). The biochemistry of apoptosis. Nature.

[40] Nakagawa T, Zhu H, Morishima N (2000). Caspase-12 mediates endoplasmic-reticulum-specific apoptosis and cytotoxicity by amyloid-beta. Nature.

[41] Pittoni P, Tripodo C, Piconese S, Mauri G, Parenza M, Rigoni A, Sangaletti S, Colombo MP (2011). Mast cell targeting hampers prostate adenocarcinoma development but promotes the occurrence of highly malignant neuroendocrine cancers. Cancer Res.

[42] Dechant MJ, Fellenberg J, Scheuerpflug CG, Ewerbeck V, Debatin KM (2004). Mutation analysis of the apoptotic ‘death-receptors’ and the adaptors TRADD and FADD/MORT-1 in osteosarcoma tumor samples and osteosarcoma cell lines. Int J Cancer.

[43] He Y, Liu F, Zhang L (2011). Growth inhibition and apoptosis induced by lupeol, a dietary triterpene, in human hepatocellular carcinoma cells. Biol Pharm Bull.

